# Metal-Catalyzed Cross-Coupling Reactions on Azaindole Synthesis and Functionalization

**DOI:** 10.3390/molecules23102673

**Published:** 2018-10-17

**Authors:** A. Sofia Santos, Ana C. Mortinho, M. Manuel B. Marques

**Affiliations:** LAQV@REQUIMTE, Departamento de Química, Faculdade de Ciências e Tecnologia, Universidade Nova de Lisboa, Campus de Caparica, 2829-516 Caparica, Portugal; asb.santos@campus.fct.unl.pt (A.S.S.); a.mortinho@campus.fct.unl.pt (A.C.M.)

**Keywords:** azaindole, metal-catalyzed, heterocycles, cross-coupling reactions

## Abstract

Azaindoles are rare in nature but extremely attractive for drug discovery programs. Azaindoles can be obtained by diverse methods, including those involving metal-catalyzed reactions. This important core has been fascinating the scientific community due to their challenging synthesis and relevant bioactivity. This paper highlights the diverse synthetic methodologies developed to date involving metal-catalyzed reaction to attain azaindoles and its functionalization.

## 1. Introduction

Azaindoles are heterocyclic structures that have enticed the interest of the scientific community, since they are bio-isosteres of the indole and considered privileged structures in medicinal chemistry. This nucleus when properly functionalized can have a wide range of medicinal applications. Thus, substituted azaindoles unlike other heterocycles, can have their properties modulated by changing the substitution pattern or the position of the endocyclic nitrogen [1].

Usually azaindoles are synthesized starting from aminopyridines followed by building up of the pyrrole ring. This approach parallels the indole synthesis from anilines, however, due to the electron-deficient nature of pyridine ring that alters the electronic properties of the conjugate system, many classic indole synthetic methods are not as efficient or just do not work, though constituting a synthetic challenge [2].

**Scheme 1:**
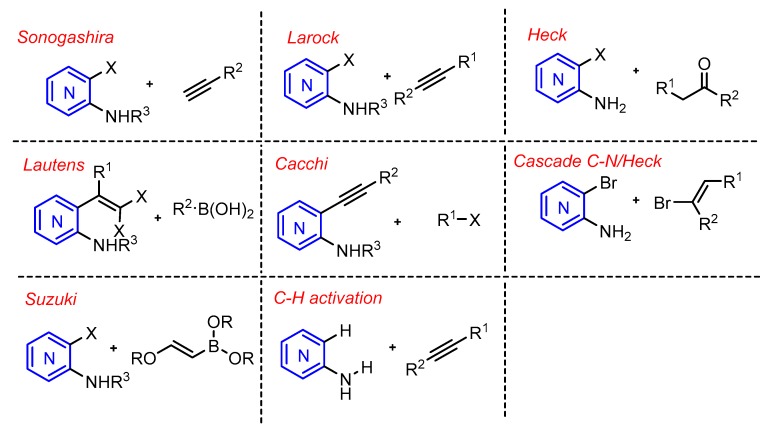
Metal-catalyzed methods reported for the construction of the azaindole core.

Metal-catalyzed cross-coupling reactions, constitute a very modern topic in organic synthesis, and are highly useful for the construction and derivation of these aminopyridine-containing heterocycles (Scheme 1). In particular, the well-known Sonogashira, Heck, and Suzuki cross-couplings have been used on the synthesis of azaindoles. Other methods involving metal-catalyzed reactions have also been described such as the Cacchi and Lautens methods. The metal-catalyzed C–H activation reaction has been scarcely explored in azaindole synthesis, as well as its functionalization.

## 2. Metal-Catalyzed Cross-Coupling Reactions

### 2.1. Sonogashira Reaction

The Sonogashira cross-coupling reaction was first reported by K. Sonogashira in 1975 and was established as a method for C–C bond formation via palladium-catalyzed coupling of terminal alkynes with aryl halides or allyl halides.

When applied to azaindole synthesis, the Sonogashira reaction usually involves amino-halopyridines that can be coupled with terminal alkynes by Sonogashira coupling, followed by ring formation, in the presence of strong bases like potassium hydride or copper-mediated cyclization.

**Scheme 2:**
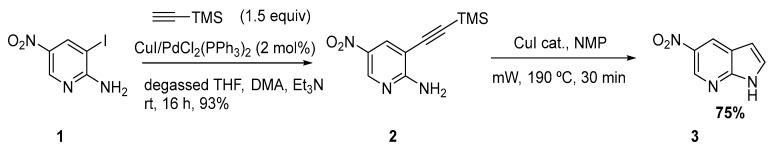
Sonogashira mediated synthesis of 5-nitro-7-azaindole [3].

In order to explore microwave assisted copper-mediated cyclization S. Pearson reported the performance of several Sonogashira reactions with nitro-substituted aminopyridines [3]. The first step involved a Sonogashira reaction of 2-amino-3-iodo-5-nitropyridine **1** with TMSA in a THF/dimethylacetamide (DMA) mixture, followed by cyclization to the azaindole structure **3** using catalytic CuI under microwave irradiation (Scheme 2).

**Scheme 3:**
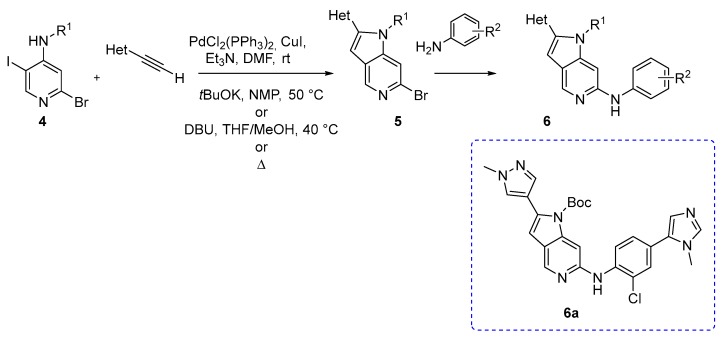
Synthesis of 5-azaindole derivatives as promising mitotic kinase monopolar spindle 1 inhibitors [4].

S. Naud and coworkers reported the synthesis of azaindole derivatives as possible inhibitors of mitotic kinase monopolar spindle 1 (MPS1), which is usually overexpressed in many human cancers. The authors used an appropriately substituted 4-amino-2-bromo-5-iodopyridine (**4**) and an alkyne, and synthesized several key intermediates using a palladium-mediated Sonogashira coupling, followed by intramolecular cyclization. These intermediates **5** were then functionalized via a C–N cross-coupling reaction (Scheme 3) [4].

Sonogashira coupling conditions were optimized according to the starting material. The Sonogashira reactions were carried using PdCl_2_(PPh_3_)_2_, CuI, and Et_3_N in DMF and the temperature was RT or 60 °C, according to the synthetic approach (Scheme 3). Compound **6a** was the most promising since it demonstrated a good oral pharmacokinetic profile in mouse and rat as well as inhibition of MPS1 activity in vivo following oral administration [4].

**Scheme 4:**
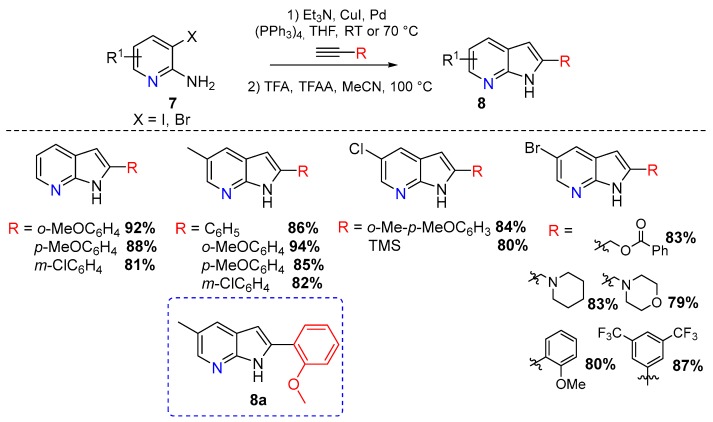
Acid-catalyzed synthesis of 7-azaindoles **8** using amino-halopyridines and amino-dihalopyridines [5].

The acid-catalyzed synthesis of 7-azaindoles was reported by T. Leboho and coworkers in 2014. The authors synthesized several 7-azaindole derivatives **8** from 3-alkynyl-2-aminopyridines (Scheme 4) [5].

The Sonogashira coupling reaction was performed using a CuI/Pd(PPh_3_)_4_ catalytic system, followed by ring closure under acidic conditions. A wide range of acids was used including HCl, H_2_SO_4_, AcOH, and trifluoroacetic acid (TFA). The best yields were obtained using 1 equiv of TFA and 1.3 equiv of TFAA in MeCN and heating to reflux for 8 h (Scheme 4). These structures were then evaluated for their antimicrobial activity with compound **8a** being the most active against *Pseudomonas aeruginosa* [5]. 

**Scheme 5:**
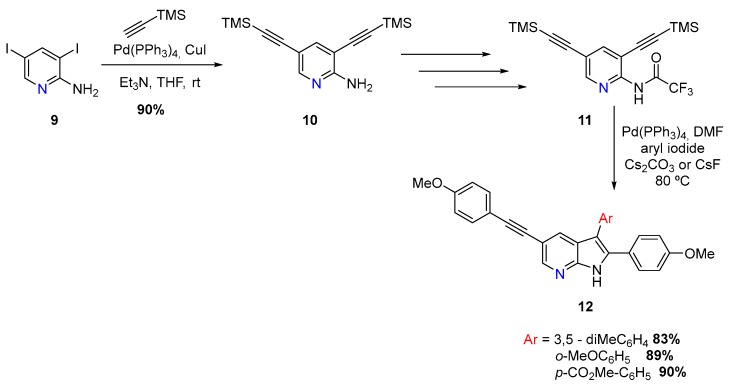
Synthesis of azaindoles **12** using double Sonogashira coupling starting from 3,5-diiodoaminopyridine **9** [6].

New 7-azaindole derivatives (**12**) were discovered as promising inhibitors for the gastrointestinal protozoal parasite *Giardia duodenalis* by T. Leboho and coworkers, via a double Sonogashira reaction, using dihalogenated aminopyridines (**9**) [6]. Two paths were explored, one starting with 3,5-diiodoaminopyridine and other with 5-bromo-3-iodoaminopyridine (Scheme 5).

The first approach involved a double Sonogashira using 3,5-diiodoaminopyridine (**9**) and a Pd(PPh_3_)_4_/CuI catalytic system, followed by removal of trimethylsilyl with TBAF. Furthermore, another double Sonogashira coupling was employed using 4-methoxyiodobenzene. This aminopyridine was then converted into the trifluoroacetamide derivative **11** and subjected to a Cacchi reaction. With the use of several aromatic iodides, and a Pd(PPh_3_)_4_ catalytic system, with Cs_2_CO_3_ or CsF as base, it was possible to synthesize three 2,3,5-trisubstituted azaindoles (**12**) (Scheme 5) [6].

**Scheme 6:**
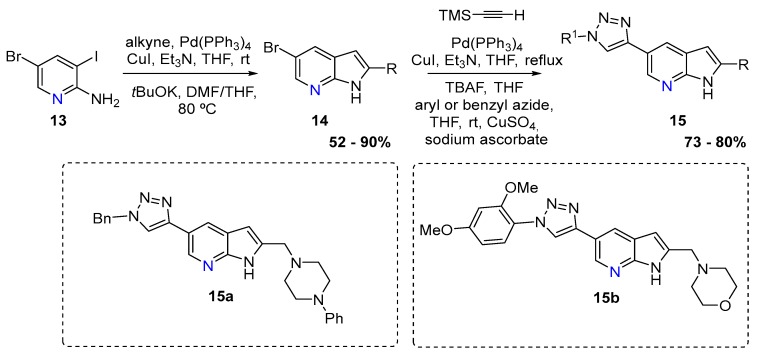
Double Sonogashira using 5-iodo-3-iodoaminopyridine (**13**) [6].

The second approach relied on a double Sonogashira using 5-bromo-3-iodoaminopyridine (**13)** that afforded several derivatives that were then treated with *t*-BuOK, DMF/THF at 80 °C. The azaindoles (**14**) were then functionalized to afford several 7-azaindoles (**15**) bearing triazole and quinoxaline moieties, with yield up to 87%. Compound **15a** and **15b** showed the best activity against the *Giardia duodenalis* trophozoites (Scheme 6) [6].

**Scheme 7:**
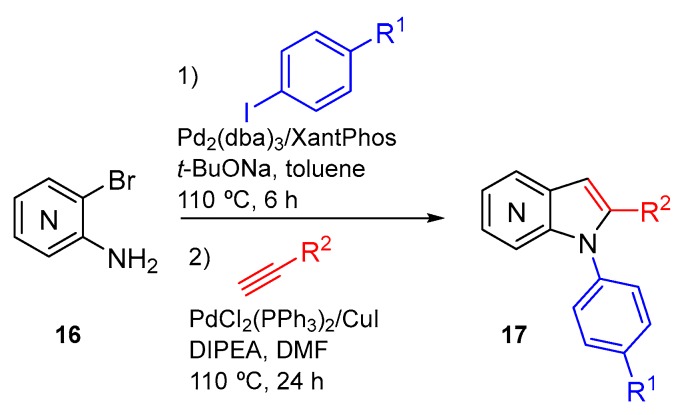
General scheme for the one-pot synthesis of 1,2-disubstituted 4-,5-,6- and 7-azaindoles **17** [7].

In 2017 we reported a one-pot approach for azaindole synthesis that involved N-arylation and Sonogashira coupling reaction followed by in situ cyclization (Scheme 7). This methodology uses amino-halopyridines as starting materials and allows the synthesis of 1,2-disubstituted 4-, 5-, 6- and 7-azaindoles [7].

**Scheme 8:**
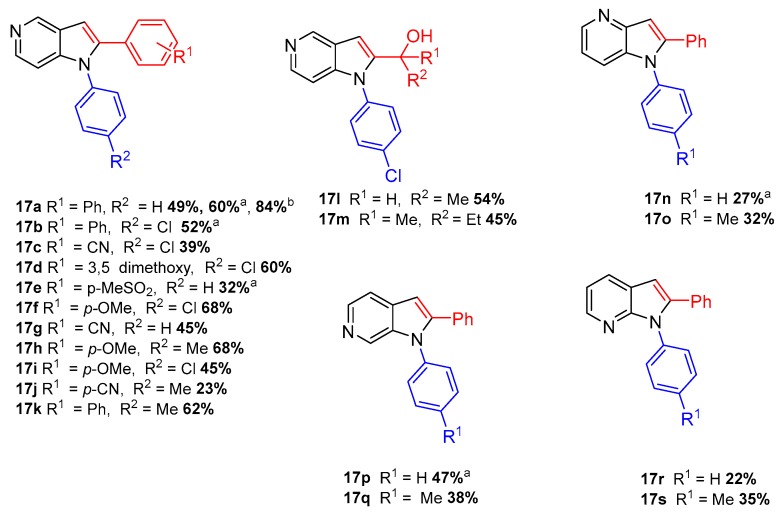
Scope of the one-pot N-arylation/Sonogashira/cyclization [7]. ^a^ Reaction carried stepwise. ^b^ Reaction carried out at 1 mmol scale.

In order to study the reaction scope, several iodides were employed in the N-arylation reaction as well as several alkynes in the Sonogashira reactions (Scheme 8). The results obtained demonstrate that this methodology exhibits a wide scope and compatibility with electron-withdrawing and electron-donating groups.

### 2.2. Larock Reaction

Recently, transition metal-catalyzed approaches to prepare azaindoles from appropriately substituted pyridines and terminal alkynes have been reported [8,9]. These include a coupling/cyclization process involving palladium or copper catalysis, an intramolecular Heck reaction of enamine derivatives [10], and a heteroannulation of internal alkynes [11,12,13,14]; according to the procedure developed by Larock for the synthesis of indoles [15,16].

**Scheme 9:**
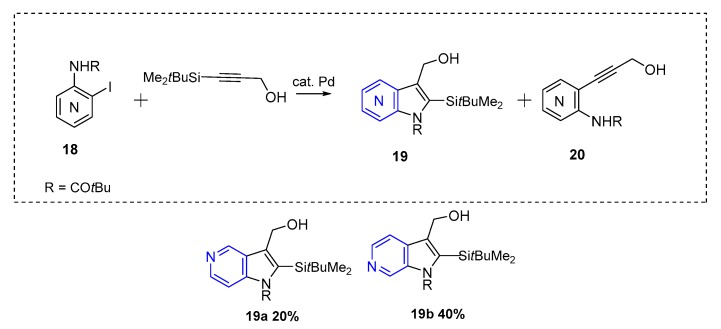
Pd-Catalyzed reactions of aryl iodides with propargyl alcohols [14]**.**

The first azaindole synthesis employing Larock methodology was reported in 1993 by Gronowitz et al. This method afforded substituted 5 and 6-azaindole (**19**) in moderate yields (up to 40% in the case of **19b**, Scheme 9) [14]. The palladium source used was Pd(OAc)_2_ (5 mol %) in the presence of KOAc as base (5 equiv).

**Scheme 10:**
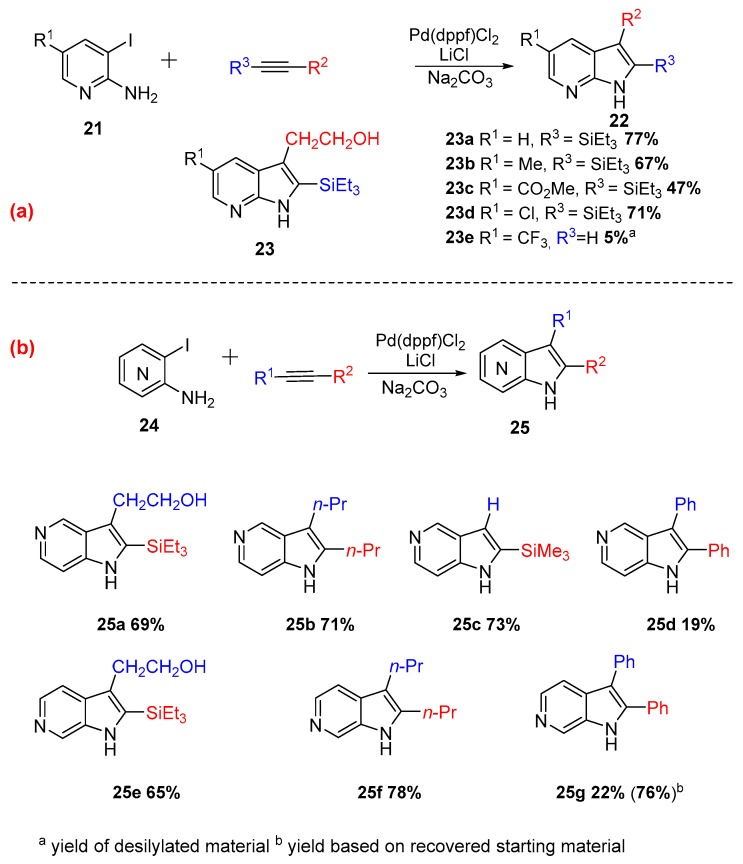
Synthesis of 2,3-disubstituted-5-azaindoles, 2,3,5-trisubstituted-7-azaindoles, and 2,3-disubstituted-6-azaindoles.

In 1998, Ujjainwalla et. al. pursued a methodology to access azaindoles substituted in the pyridine ring. This method gave access to 2,3,5-trisubstituted-7-azaindoles (Scheme 10a), 2,3-disubstituted-5-azaindoles (Scheme 10b), and 2,3-disubstituted-6-azaindoles (Scheme 10b) with very good yields (up to 77%). The catalytic system was changed to Pd(dppf)Cl_2_ rather than Pd(OAc)_2_, affording higher regioselectivity, reproducibility, and improved yield [12].

**Scheme 11:**
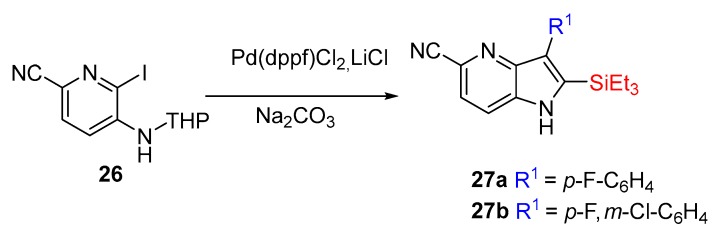
Synthesis of 2,3-disubstituted-4-azaindoles **27**.

Inspired by these discoveries, H. Koolman et al. executed a complementary route employing Larock methodology to synthesize a tyrosine kinase inhibitor, a 4-azaindole core attached to a diaryl substitution in the C-2 and C-3 position of **27** (yields from 48 to 66%, over two steps). These products were not isolated since they were a part of an extensive synthesis (Scheme 11) [17].

**Scheme 12:**
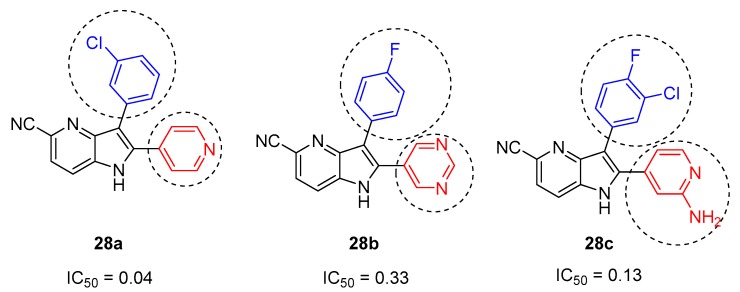
Important bioactive compounds **28a**, **28b**, and **28c** and their respective IC_50_.

The objective of these studies was the synthesis of the compounds **28a**, **28b**, and **28c** (Scheme 12) in order to measure their inhibitory activity of c-Met (tyrosine-protein kinase Met).

### 2.3. Heck Reaction

In 1999, the first synthesis of azaindoles via Heck reaction was demonstrated by Blache and coworkers [18]. This approach consisted of enamine formation in the presence of Pd(Ph_3_)_4_ and NaHCO_3_ in HMPA at 140 °C, consisting of a Hegedus–Mori–Heck reaction. However, the reaction only led to low yields and high amounts of recovered starting enamines from 2-aminopyridine.

**Scheme 13:**
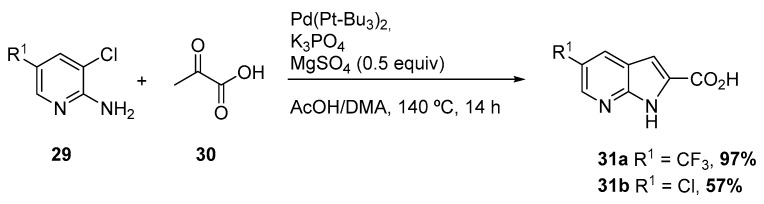
Synthesis of 7-azaindole via enamine formation and subsequent Heck reaction [19]**.**

Later, in 2004, Nazaré and coworkers reported a one-step palladium-catalyzed annulation procedure for the synthesis of substituted, polyfunctionalized 4- and 7-azaindoles **31**, by reaction of amino *ortho*-chloropyridines **29** with a variety of pyruvic acid derivatives **30**, under mild conditions (Scheme 13) [19]. The protocol involved an enamine formation followed by Heck reaction. The protocol consisted on the treatment of a functionalized 2-amino *ortho*-chloropyridine with 3 equiv of an acyclic ketone in the presence of Pd(P*t*-Bu_3_)_2_, a base, and MgSO_4_ as a water scavenger. The method revealed to be applicable both for indoles and azaindoles, starting from the corresponding *ortho*-chloro anilines and amino *ortho*-chloropyridines, respectively.

**Scheme 14:**
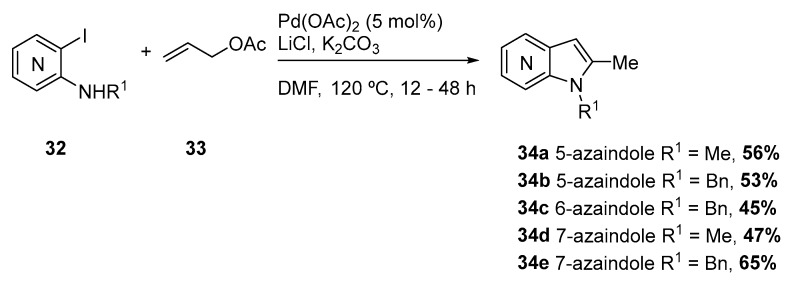
Synthesis of 2-methyl 5-, 6-, and 7-azaindoles **34** via palladium-catalyzed annulation of amino-*ortho*-iodopyridines **32** with allyl acetate **33** [20].

The synthesis of 2-methyl 5-, 6-, and 7-azaindoles (**34**) via palladium-catalyzed annulation was reported by Yum et al. that described the reaction of *ortho*-iodoarylamines (**32**) with allyl acetate under Pd(OAc)_2_ (5 mol %), LiCl (1 equiv), K_2_CO_3_ (3 equiv), allyl acetate (**33**) (and 2 equiv) in DMF at 120 °C [20]. The authors extended their protocol to other aromatic ring fused pyrrole derivatives with several *ortho*-iodoarylamines (**32**) with allyl acetate (**33**) under the optimized reaction conditions, such as indoles and pyrrolo-quinolines. Higher yields were obtained when N-protected substrates were used however, the azaindoles were obtained in moderate yields (Scheme 14).

The authors proposed that the mechanism of the reaction proceeds via formation of a π-allyl complex followed by intermolecular nucleophilic attack generating the pyrrole ring and regenerating Pd(0).

**Scheme 15:**
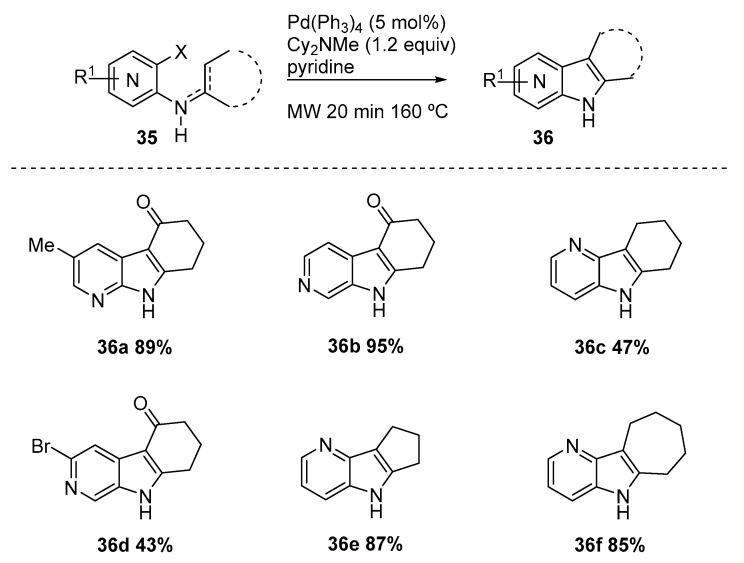
Synthesis of azaindoles (**36**) via an intramolecular Heck reaction under microwave conditions [21].

An intramolecular Heck reaction (Hegedus–Mori–Heck reaction) was reported for the preparation of several azaindoles by Lachance and coworkers [21]. The authors described the intramolecular Heck reaction of imines/enamines (**35**) under microwave conditions in the presence of Pd(Ph**_3_**)**_4_** and Cy**_2_**NMe in pyridine, and obtained good yields of azaindoles (**36**) (Scheme 15). The protocol revealed to be compatible with the presence of bromine, ketone, and esters. All isomers of azaindoles were prepared directly from the corresponding amino-*ortho*-halogenated pyridines (halogen= iodo, bromo, and chloro) in the presence of a cyclic ketone or an acyclic aromatic ketone, in up to 80% yield. The use of microwave irradiation allowed to reduce the reaction time and promoted the palladium coupling.

**Scheme 16:**
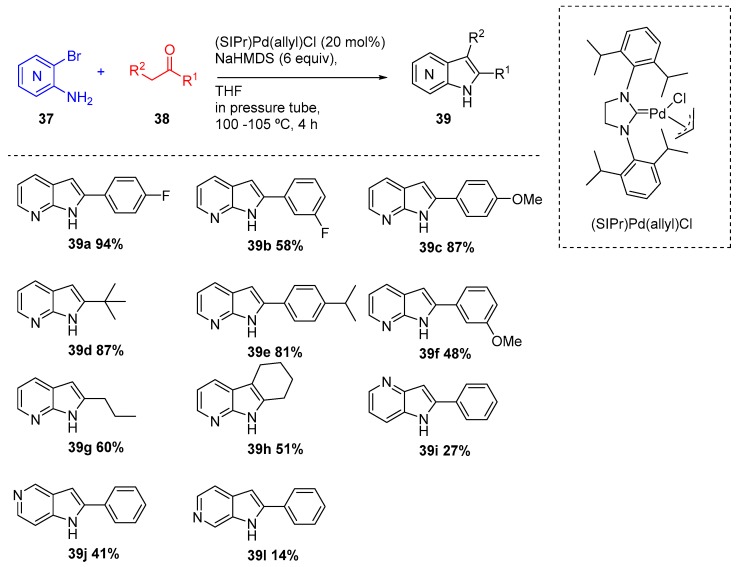
Synthesis azaindoles (**39**) from ketones (**38**) and amino-*ortho*-halopyridines (**37**).

In 2010, Spergel and coworkers reported a one-pot method for the construction of a variety of azaindoles (**39**) using simple ketones (**38**) and amino-*ortho*-halopyridines (**37**) via a palladium-catalyzed α-heteroarylation of ketone enolates (Scheme 16) [22]. A N-heterocyclic carbene palladium catalyst, the (SIPr)Pd(allyl)Cl described by Nolan [23], was examined and revealed to be suitable to convert the amino-*ortho*-bromopyridines (**37**) in the corresponding azaindoles (**39**) in low to high yields. The method allowed preparation of a variety of azaindoles (all regioisomers) from unsymetric ketones.

### 2.4. Suzuki Coupling and Lautens Reaction

**Scheme 17:**
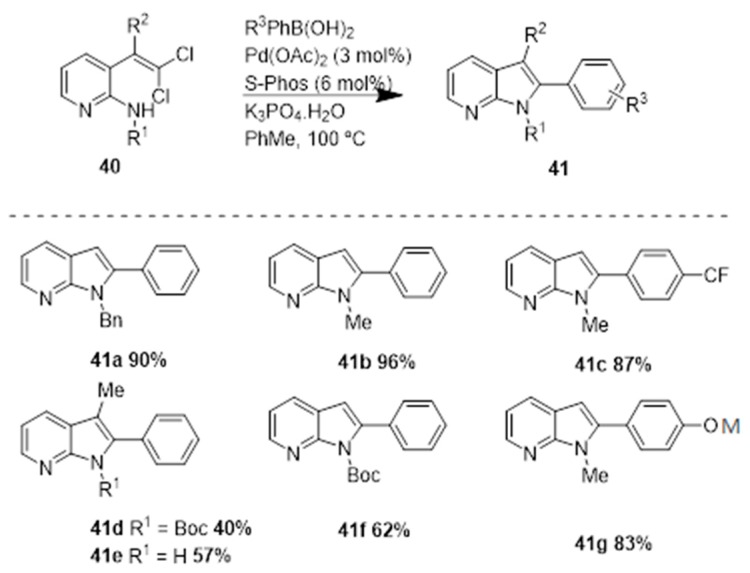
Synthesis of azaindoles (**41**) by a Pd-catalyzed reaction of *gem*-dichloroolefins and a boronic acid, via a tandem intramolecular C–N and intramolecular Suzuki coupling process [24].

In 2007, Mark Lautens and coworkers [24] reported a palladium-catalyzed reaction of *gem*-dichloroolefins and a boronic acid via a tandem intramolecular C-N and intramolecular Suzuki coupling process. The group initiated their studies with indole synthesis via a tandem C-N/Suzuki coupling of *gem*-dibromovinylaniline. However, the authors did not observe 7-azaindole formation under these conditions when a dibromovinyl aminopyridine was used as substrate, presumably due to catalyst poisoning. To overcome this problem, the authors used N-protected substrates, that led to successful reactions; the best yields were obtained with N-alkyl substrates (N-Me), e.g., **41b**, **41c**, and **41g** were obtained in high yields (Scheme 17).

**Scheme 18:**
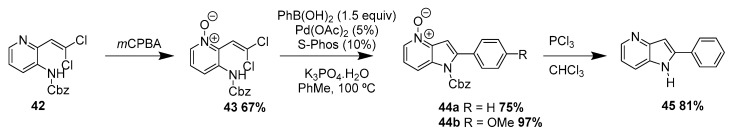
Synthesis azaindole 45 by a Pd-catalyzed reaction of *gem*-dichloroolefins and a boronic acid using the pyridyl N-oxide 43 as substrate [24].

The authors also prepared 6-azaindoles using N-Boc protected substrates. Concerning the 5-azaindoles, the authors observed formation of the bis-Suzuki coupling product along with the desired product. Thus, in order to avoid the formation of this mixture the authors reverted the properties of the substrate by using the pyridyl group protected as a N-oxide. A complex mixture of products was also observed by the authors while preparing the 4-azaindoles, this might be due to the coordination of the pyridyl nitrogen that retards the C–N bond formation. The authors overcame this problem by protecting the pyridyl nitrogen as the N-oxide (**43**), and observed the formation of the corresponding products in good to high yields of **44a** and **44b**, respectively (Scheme 18). This approach added to more steps in the synthetic sequence, and the final azaindole (**45c**) was obtained in 81% yield. This method represents a very flexible protocol to access all four isomers of azaindole in good to excellent yield.

The Suzuki reaction was also applied to the construction of azaindoles by Swen Hoelder and coworkers [25]. The authors described an efficient two-step route, starting from chloroamino-*N*-heterocycles, to prepare a wide range of aza- and diazaindoles, avoiding the use of protecting groups. The method involved an optimized Suzuki–Miyaura coupling with (2-ethoxyvinyl)borolane using 3 mol % of SPhos/Pd(OAc)_2_ (2.5:1) as catalyst in refluxing MeCN/H_2_O (3:2) with K_3_PO_4_ as base, followed by acetic acid-catalyzed cyclization.

### 2.5. Cacchi Reaction

**Scheme 19:**
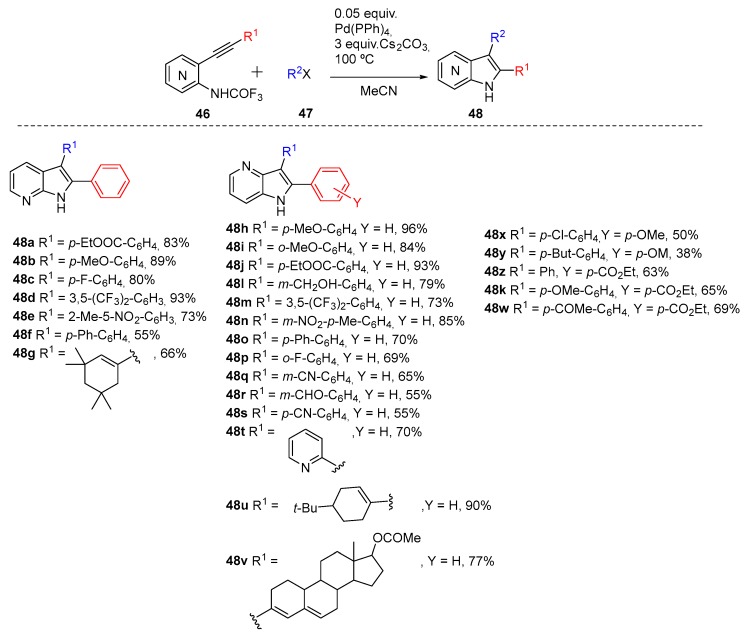
Aminopalladation-reductive elimination reaction for 4- and 7-azaindole synthesis (**48**) from 2-Alkynyl-3-trifluoroacetamidopyridines 46.

In 2005, Cacchi et al. adapted their protocol that accessed indoles to build the azaindole core [26]. The procedure describes an aminopalladation-reductive elimination protocol to develop a solution-phase synthesis of free N-H 2,3-disubstituted azaindoles. Previously the authors showed that the basicity of the nitrogen of the starting acetanilides plays a crucial role in the synthesis of indoles, via the aminopalladation-reductive elimination process [27]. Furthermore, the pyridine moiety plays a beneficial role in favoring the formation of the free N–H pyrrole ring, since when o-(phenylethynyl)acetanilide was subjected to the same cyclization conditions the starting alkyne was recovered in 98% yield. Therefore, they examined the use of the trifluoroacetamido derivative (**46**), which produced the corresponding azaindole (**48**) in 3.5 h under the same conditions, confirming the crucial role of the trifluoroacetyl group in this type of cyclization (Scheme 19).

### 2.6. C-H Activation Reaction

This reaction constitutes a great advance in metal-catalysis, however there are still some challenges, such as the regioselectivity [28] (C–H sp2 and sp3 bonds are ubiquous), the low reactivity due to a great energy barrier to break a C–H bond (104 kcal/mol) and at last the chemoselectivity [29,30].

**Scheme 20:**
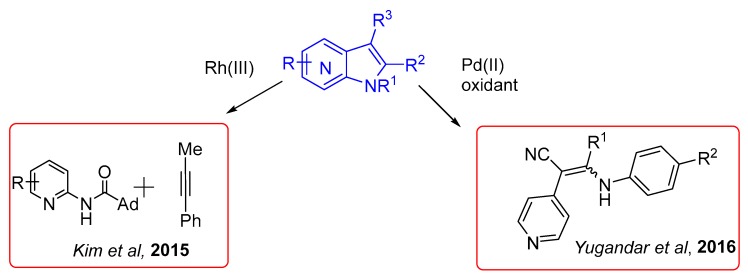
C–H activation to access the azaindole core.

Regarding azaindole synthesis, there are still few reports on C–H activation reaction applied to afford these compounds. One of such approaches relies on the use of rhodium and palladium catalysis affording the 7-azaindole isomer (Scheme 20).

Organic synthesis has changed hugely mainly due to the introduction of metal-catalyzed reactions. However, these approaches require the presence of reactive functional groups in one of both coupling partners. Consequently, the reducing number of necessary functionalities in coupling reactions, like in the case of C–C coupling thorough C–H activation, emerged as an attractive alternative [31,32,33].

#### 2.6.1. C–H via Rhodium Catalysis

The low reactivity of aminopyridines complicates the building up of the azaindole ring. These difficulties make substrate prefunctionalization crucial in synthetic methods such as Larock. These methods require the use of amino *ortho*-halogenated pyridines to access different substitution patterns.

**Scheme 21:**
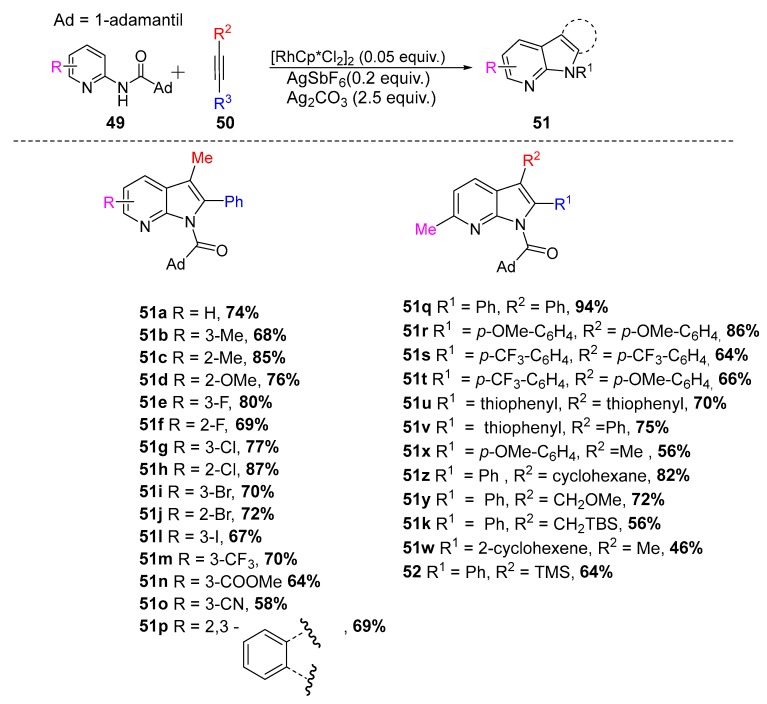
Synthesis of azaindoles (**51**) from alkynes and N-substituted aminopyridines (**49**) using Rh(III) catalysis [34].

In 2015, Kim et al. created a strategy envisioning C–H activation with rhodium(III) catalysis. This approach was based in the use of aminopyridines and alkynes. Since the aminopyridine ring can be considered a Lewis base, a Lewis acid (Ag_2_CO_3_) was strategically used to coordinate with the N atom of the pyridine ring, facilitating the annulation process (Scheme 21) [34].

**Scheme 22:**
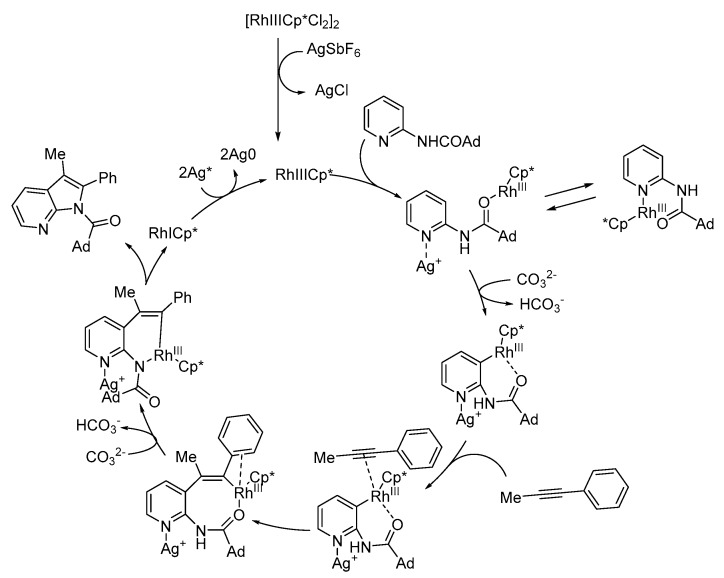
Plausible reaction pathway of Rh(III) catalysis for azaindole synthesis [34].

The authors proposed a plausible mechanism for the rhodium-catalyzed 7-azaindole synthesis, as depicted in Scheme 22. The authors propose that the silver ions coordinate to the pyridyl nitrogen atom and facilitate the C–H bond cleavage of the aminopyridine.

#### 2.6.2. C-H Activation Reaction via Palladium Catalysis

**Scheme 23:**
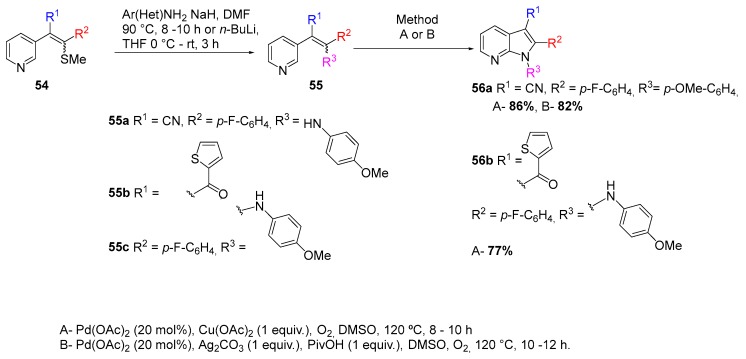
Synthesis of 7-azaindole core from 2,3-(het)aryl-3-*N*-aryl/acylenaminonitriles and enaminones.

Recently, following Buchwald’s pioneering report of carbazole synthesis via Pd(II)-catalyzed intramolecular C–H activation/C–N bond formation, attention has been turned on the C–H functionalization reactions for the construction of various heterocyclic ring systems [35,36,37]. These methods have also been applied to the indole synthesis and intramolecular cross dehydrogenative coupling (CDC) has become a promising protocol for the synthesis of indoles from enamines and imines involving C3–C3a bond formation [38,39,40,41,42,43,44]. In continuation of these studies, Yugandar et al. disclosed an efficient route to 1-*N*-aryl/NH-2-(het)aryl/alkyl-3-cyano/aroylindoles and their heterofused analogs by palladium-catalyzed intramolecular oxidative C–H functionalization–amination of readily available 2,3-(het)aryl-3-*N*-aryl/acylenaminonitriles and enaminones (Scheme 23) [45].

This reaction displays high regioselectivity and good functional group tolerance at various positions of azaindole skeleton along with high yields in this cyclization reaction. The reaction represents one of the few examples, in which an aryl C–H bond is activated by an aminoaryl directing group, that subsequently acts as the reaction partner in the same process [46].

## 3. Conclusions

Azaindoles can be prepared from aminopyridines, similarly to the indole synthesis from anilines. The most recent synthetic approaches rely on the use of metal-catalyzed reactions. Metal-catalyzed cross-coupling reactions, which constitute a very modern and emergent topic in organic synthesis, can be highly useful for the construction and derivation of these aminopyridine-containing heterocycles. However, the challenging nature of aminopyridines difficults the application of metal-catalyzed reactions. Despite this, great advances have been achieved on the use of metal-catalyzed reactions to prepare and functionalyse azaindoles. The main difficulty is to develop a procedure that is wide in scope, allowing access to all isomers; is high yielding; uses mild conditions; and avoids the use of protecting groups, especially at the amino group.

So far several synthetic routes have been reported for the preparation of azaindoles from aminopyridines, including Sonogashira, Larock-type, Heck, Lautens, Suzuki coupling, Cacchi, and more recently C–H activation. These methods constitute a major advance in the development of new synthetic methods to attain azaindoles.

It is expected that, in a near future, new approaches consisting of one-pot protocols involving metal-catalyzed reactions will emerge to improve the synthesis of functionalized azaindoles having a high impact in industry.
